# Handgrip strength and subclinical carotid atherosclerosis in relation to platelet levels among hypertensive elderly Japanese

**DOI:** 10.18632/oncotarget.20618

**Published:** 2017-09-01

**Authors:** Yuji Shimizu, Shimpei Sato, Jun Koyamatsu, Hirotomo Yamanashi, Mako Nagayoshi, Koichiro Kadota, Shin-Ya Kawashiri, Keita Inoue, Yasuhiro Nagata, Takahiro Maeda

**Affiliations:** ^1^ Department of Community Medicine, Nagasaki University Graduate School of Biomedical Sciences, Nagasaki, Japan; ^2^ Department of Cardiovascular Disease Prevention, Osaka Center for Cancer and Cardiovascular Disease Prevention, Osaka, Japan; ^3^ Department of Island and Community Medicine, Nagasaki University Graduate School of Biomedical Sciences, Nagasaki, Japan; ^4^ Center for Comprehensive Community Care Education, Nagasaki University Graduate School of Biomedical Sciences, Nagasaki, Japan

**Keywords:** atherosclerosis, handgrip, hypertension, platelet, sarcopenia, Gerotarget

## Abstract

Age-related disruption of microvascular endothelium exacerbates hypertension and sarcopenia; and atherosclerosis is a well-known biological response to vascular endothelial injury. Therefore, prevalence of atherosclerosis among hypertensive elderly subjects may partly indicate the presence of an appropriate response to endothelial injury. We conducted a cross-sectional study of 795 elderly hypertensive Japanese subjects aged 60-89 years. Since platelet level is an indicator of vascular repair activity, subjects were stratified by platelet counts. No significant association between handgrip strength and subclinical carotid atherosclerosis (carotid intima-media thickness (CIMT) ≥1.1mm) was observed for subjects with lower platelet counts, while a significant positive association was observed for subjects with higher platelets. Adjusted odds and 95% confidence intervals of subclinical carotid atherosclerosis for 1 standard deviation increments in handgrip strength were 0.86 (0.61, 1.22) for subjects with lower platelets and 1.82 (1.26, 2.64) for subjects with higher platelets. A positive association between handgrip strength and subclinical carotid atherosclerosis exists in hypertensive elderly subjects with higher, but not lower, platelet counts. These results lead us to speculate that subjects with a beneficial influence on prevention of sarcopenia (maintenance of handgrip strength) may possess the capacity of active endothelial repair that causes atherosclerosis.

## INTRODUCTION

Hypertension and sarcopenia are known as age-related diseases, and such diseases are reported to be exacerbated by disruption of the micro-vascular endothelium and impairment of blood flow *via* an increase in age-related inflammatory agents [1].

Endothelial dysfunction has been recognized as one of the initial mechanisms leading to atherosclerosis (increased arterial stiffness) [2]. Therefore, age-related diseases also may cause atherosclerosis. A longitudinal study of 195 older men during follow-up reported that atherosclerosis, as measured by carotid intima-media thickness (CIMT), is related to lower handgrip strength [3].

On the other hand, recent studies have reported an association between bone metabolism and vascular homeostasis [4-11] in view of the fact that hematopoietic stem cells (immature cells such as CD34-postive cells) derived from the bone marrow play a major role in vascular homeostasis [5-7]. Additionally, platelets play an important role in vascular culture together with CD34-positive cells [12-21], and also induce the differentiation of human CD34-positive cells into foam cells [20], which are a well-known contributing factor in the development of atherosclerotic lesions. In fact, CD34-positive cells have previously been observed in atherosclerotic lesions in humans [22, 23]. And platelet-derived angiogenesis regulators promote angiogenesis during wound healing, tumor growth, and response to ischemia [24], while platelet-rich plasma also promotes angiogenesis [25].

In addition, active hematopoietic bone marrow declines with age [26-29], which could result in the age-related decline of vascular homeostasis activity (including the increased capacity for developing atherosclerosis). Therefore, the presence of atherosclerosis among elderly hypertensive subjects may partly indicate the presence of active repair against disrupted micro-vascular endothelium and impaired blood flow. Since handgrip strength is an efficient tool to evaluate the loss of skeletal muscle mass and function due to its use as a predictor of old age disability [30], among elderly hypertensive subjects, those who can maintain handgrip strength with a beneficial influence on preventing sarcopenia may possess a higher capacity for developing atherosclerosis.

To clarify these associations, we conducted a cross-sectional study of 795 elderly Japanese subjects aged 60-89 years who underwent an annual health check-up from 2015-2016.

## RESULTS

Among the total study population, 394 subjects had lower platelet values (platelets < 21.6×10^4^ /μL for men and < 22.6×10^4^ /μL for women) and 401 had higher values (platelets 21.6×10^4^ /μL ≤ for men and 22.6×10^4^ /μL ≤ for women).

Table 1 shows platelet level specific characteristics of the present study population based on handgrip strength tertiles. Subjects with lower platelets demonstrated an inverse association between handgrip strength and age, mean CIMT, and a positive association with diastolic blood pressure, while subjects with higher platelet counts showed an inverse association between handgrip strength and age and a positive association with diastolic blood pressure, current smoker status and triglycerides.

**Table 1:** Platelets level-specific characteristics of study population by handgrip strength level tertiles

	Handgrip strength tertiles			
	T1 (low)	T2	T3 (high)	p
Lower platelets count				
No. at risk	123	142	129	
Age, years	77.1 ± 7.3	74.0 ± 6.5	69.4 ± 5.8	<0.001
Male, %	59.3	54.9	57.4	0.768
Systolic blood pressure, mmHg	152 ± 12	154 ± 14	151 ± 11	0.071
Diastolic blood pressure, mmHg	84 ± 10	87 ± 11	91 ± 9	<0.001
Body mass index, kg/m^2^	23.8 ± 3.7	23.8 ± 3.3	23.9 ± 3.1	0.932
Current drinker, %	58.5	52.8	53.5	0.606
Current smoker, %	1.6	6.3	7.0	0.107
Serum triglycerides (TG), mg/dL	96 ± 47	101 ± 50	103 ± 62	0.591
Serum HDL-cholesterol (HDL), mg/dL	59 ± 15	57 ± 15	61 ± 17	0.222
SerumHbA1c, %	5.7 ± 0.6	5.6 ± 0.4	5.6 ± 0.6	0.358
Mean carotid intima-media thickness (CIMT), mm	0.75 ± 0.15	0.73 ± 0.14	0.69 ± 0.12	<0.001
Higher platelets count				
No. at risk	149	124	128	
Age, years	76.1 ± 7.4	73.1 ± 6.8	68.2 ± 5.9	<0.001
Male, %	56.4	55.6	57.0	0.976
Systolic blood pressure, mmHg	153 ± 12	152 ± 13	151 ± 14	0.387
Diastolic blood pressure, mmHg	84 ± 10	86 ± 10	91 ± 9	<0.001
Body mass index, kg/m^2^	23.4 ± 3.0	23.3 ± 2.9	24.1 ± 2.8	0.053
Current drinker, %	57.0	47.6	50.1	0.136
Current smoker, %	0.0	0.8	18.0	<0.001
Serum triglycerides (TG), mg/dL	115 ± 57	112 ± 57	136 ± 102	0.022
Serum HDL-cholesterol (HDL), mg/dL	60 ± 15	58 ± 16	59 ± 16	0.789
SerumHbA1c, %	5.7 ± 0.4	5.9 ± 0.7	5.7 ± 0.5	0.015
Mean carotid intima-media thickness (CIMT), mm	0.72 ± 0.15	0.73 ± 0.16	0.71 ± 0.13	0.479

Handgrip strength levels tertiles: <25.5 kg, 25.5-35.0 kg, and >35.0kg for men and <18.5kg, 18.5-22.0kg, and >22.0kg for women. Lower platelets are defined as < 21.6×10^4^ /μL for men and < 22.6×10^4^ /μL for women.

Table 2 shows ORs and 95% CIs for carotid atherosclerosis in total subjects, stratified by platelet levels. No significant association between handgrip strength and carotid atherosclerosis was seen in subjects with lower platelet levels, whereas a significant positive association was observed for subjects with higher platelet levels. The multivariable ORs and 95% CIs of carotid atherosclerosis for 1 SD increments in handgrip strength (9.4 kg for men and 5.0 kg for women) were 0.86 (0.61, 1.22) for subjects with lower platelets and 1.82 (1.26, 2.64) for subjects with higher platelets.

**Table 2:** Odds ratios (OR) and 95% confidence intervals (CI) for carotid atherosclerosis

	Handgrip strength tertiles				1 SD increment in handgrips strength
	T1 (low)	T2	T3 (high)	*p* for trend	
Total subjects					
No. at risk	272	266	257		
No. of cases (percentage)	44 (16.2)	45 (16.9)	37 (14.4)		
Sex-and age-adjusted OR	1.00	1.32 (0.82, 2.11)	1.52 (0.89, 2.63)	0.117	1.20 (0.96, 1.50)
Multivariable OR	1.00	1.32 (0.81, 2.16)	1.58 (0.89, 2.80)	0.111	1.23 (0.97, 1.56)
Lower platelets count					
No. at risk	123	142	129		
No. of cases (percentage)	29 (23.6)	20 (14.1)	15 (11.6)		
Sex-and age-adjusted OR	1.00	0.63 (0.33, 1.20)	0.65 (0.30, 1.38)	0.208	0.78 (0.56, 1.08)
Multivariable OR	1.00	0.75 (0.38, 1.50)	0.78 (0.35, 1.74)	0.505	0.86 (0.61, 1.22)
Higher platelets count					
No. at risk	149	124	128		
No. of cases (percentage)	15 (10.1)	25 (20.2)	22 (17.2)		
Sex-and age-adjusted OR	1.00	3.10 (1.50, 6.41)	4.00 (1.76, 9.10)	<0.001	1.80 (1.29, 2.51)
Multivariable OR	1.00	2.82 (1.30, 6.11)	3.75 (1.54, 9.17)	0.004	1.82 (1.26, 2.64)

Multivariable OR: adjusted further for age and sex, systolic blood pressure, body mass index, smoking status, alcohol intake, serum triglycerides, serum HDL-cholesterol, and HbA1c. Handgrip strength levels tertiles: <25.5 kg, 25.5-35.0 kg, and >35.0kg for men and <18.5kg, 18.5-22.0kg, and >22.0kg for women. Lower platelets are defined as < 21.6×10^4^ /μL for men and < 22.6×10^4^ /μL for women. Carotid atherosclerosis is defined as a carotid intima-media thickness ≥1.1mm. SD: standard deviation. 1 SD increment in handgrips strength are 9.4kg for men and 5.0kg for women.

An investigation into the effects of the association between handgrip strength and the two platelet level categories (high and low) on carotid atherosclerosis revealed a significant interaction; p values for the effect of this interaction were *p* = 0.002 for the sex-and age-adjusted model and *p* = 0.002 for fully adjusted model.

To verify the validity of using a sex-combined model in the present study, we also conducted a sex-specific analysis, and found essentially the same associations. Among subjects with lower platelets, the multivariable ORs and 95% CI of carotid atherosclerosis for 1 SD increments in handgrip strength were 1.20 (0.77, 1.85) for men and 0.47 (0.25, 0.87) for women, while among subjects with higher platelets, the corresponding values were 2.04 (1.16, 3.58) for men and 1.55 (0.92, 2.62) for women.

## DISCUSSION

The major findings of the present study reveal a positive association between handgrip strength and carotid subclinical atherosclerosis among hypertensive subjects with higher, but not lower, platelet counts. These results lead us to speculate that subjects with a beneficial influence on prevention of sarcopenia (maintenance of handgrip strength) may possess the capacity of active endothelial repair that causes atherosclerosis.

Sarcopenia is associated with impairment of capillary function [31]; and microvascular endothelium disruption and impaired blood flow could be exacerbated by common age-related diseases, including hypertension and sarcopenia [1].

Our previous study of Japanese elderly men reported a positive association between circulating CD34-positive cells and handgrip strength in hypertensive subjects [32]. We therefore speculate that vascular maintenance provided by circulating CD34-positive cells is one possible background mechanism behind the positive association between handgrip strength and carotid subclinical atherosclerosis.

Recently, platelets have been revealed to play an important role in vascular culture maintenance together with CD34-positive cells [12-21]. Platelets induce the differentiation of human CD34-positive cells not only into endothelial cells but into foam cells as well [20], which are a well-known contributing factor in the development of atherosclerotic lesions. Previously, we reported that among subjects with hypertension, platelet count is positively associated with CIMT but not with circulating CD34-positive cells, since consumptive reduction of circulating CD34-positive cells is induced by aggressive vascular repair [33]. Therefore, among hypertensive subjects, those with a higher platelet count might have higher vascular repair activity than those with lower platelet counts.

Since hematopoietic stem cells, known as CD34-positive cells, promote angiogenesis [5], development of atherosclerosis may also indicate vascular culture maintenance activity, including microvascular endothelium repair and collateral blood flow, which plays an important role in providing blood flow to muscles.

On the other hand, active hematopoietic bone marrow declines with age [26-29], which may result in insufficient vascular endothelium repair in elderly subjects. Since atherosclerosis is a result of aggressive endothelial repair, the existence of atherosclerosis in hypertensive elderly subjects also partly indicates elevated bone marrow activity, which reduces the risk of insufficient vascular endothelium repair and microvascular endothelium disruption. With these mechanisms, handgrip strength should be positively associated with atherosclerosis.

Among elderly hypertensive subjects, while clinical atherosclerosis is known to be associated with blood flow impairment, subclinical atherosclerosis may be associated with comparative active maintenance of muscle blood flow by indicating endothelial repair activity.

In addition to that, since capillary network remodeling such as angiogenesis is essential for the physical adaptation of skeletal muscle to exercise, occurs in response to the mechanical forces of elevated capillary share stress and cell stretch [34, 35] the status of maintenance handgrip also should stimulate bone marrow activity.

However, the necessity of endothelial repair, which stimulates bone marrow activity, is determined by the degree of endothelial injury. Subjects with lower levels of endothelial injury might therefore have lower platelet values and a stronger handgrip. Therefore, in subjects with a lower platelet count, no significant positive association between handgrip strength and atherosclerosis would be observed.

**Figure 1:**
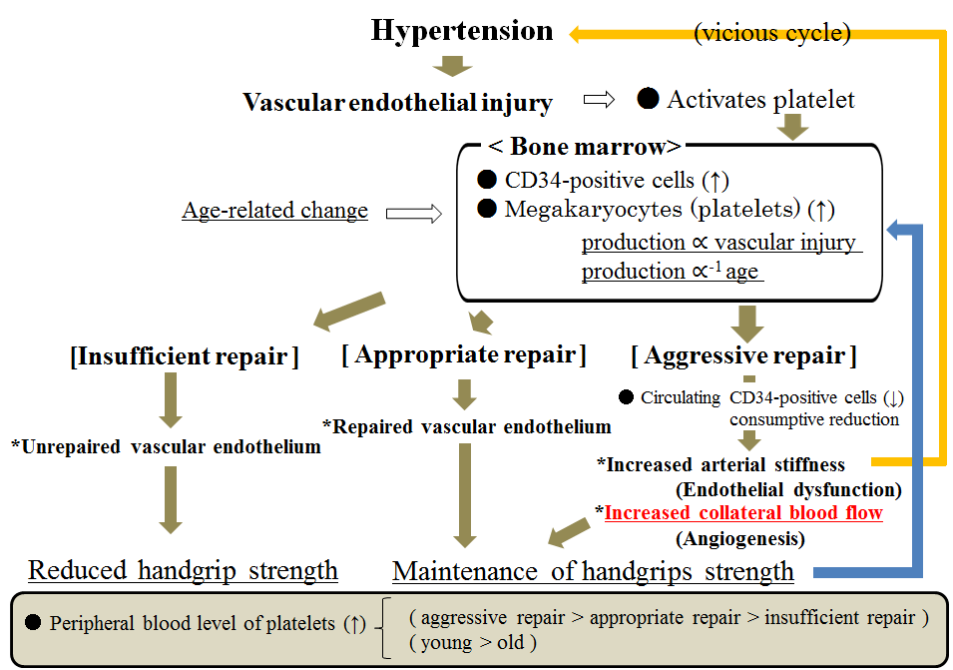
Possible mechanism underlying the association between handgrip strength and endothelium repair among hypertensive subjects

Figure 1 shows the possible mechanism underlying the association between handgrip strength and endothelium repair among hypertensive subjects. Higher platelet counts indicate aggressive endothelial repair that results in the development of atherosclerosis, angiogenesis and maintenance of the microvascular endothelium, which is associated with maintaining handgrip strength. And lower platelet counts result in two types of subjects—those who possess sufficient repair capacity and present with less damage to the endothelium, and those with insufficient repair of the endothelium due to the age-related reduction of bone marrow activity. Since hypertension and atherosclerosis have a bidirectional association (vicious cycle) [36], the favorable association between maintaining handgrip strength and formation of atherosclerosis is especially true for subjects with hypertension. The present study found a significant effect on carotid atherosclerosis from handgrip strength and two platelet level categories (high and low), which partly supports the above-mentioned different characteristics on endothelial repair capacity based on platelet levels.

Potential limitations of this study warrant consideration. Although a significant positive association exists between handgrip strength and CIMT, no data was available with regard to the evaluation of endothelial function. Further analyses that include endothelial function-related data such as Flow Mediated Dilation (FMD) will be necessary. And because platelets play an important role in vascular culture maintenance together with CD34-positive cells [12-21], further analyses that include CD34-positive cell data may be informative in clarifying the background mechanisms behind the present results. Additionally, as this was a cross-sectional study, causal relationships were not able to be established.

In conclusion, a positive association exists between handgrip strength and subclinical carotid atherosclerosis among elderly hypertensive subjects with higher, but not lower platelet counts. These results lead us to speculate that subjects with a beneficial influence on prevention of sarcopenia (maintenance of handgrip strength) may possess the capacity of active endothelial repair that causes atherosclerosis.

## MATERIALS AND METHODS

### Study population

The study population comprised 1,803 residents aged 60-89 years from the western rural communities of Goto city who had undergone an annual medical check-up from 2015 to 2016 as recommended by the Japanese government. Subjects without blood pressure data (*n* = 2) were excluded from the analysis. Endothelial repair activity evaluated by circulating CD34-positive cell count may be essential for maintaining handgrip strength in elderly hypertensive subjects [32]. Since platelets play an important role in vascular culture together with CD34-positive cells [12-21], and platelet count is positively associated with CIMT in hypertensive subjects but not in non-hypertensive subjects [33], subjects without hypertension were excluded (*n* = 910). An analysis limited to hypertensive subjects should emphasize the impact of atherosclerosis as an indicator of endothelial repair, which relates to maintaining handgrip strength. We defined hypertension according to previous studies that evaluated the impact of platelets and circulating CD34-positive cell count as an indicator of endothelial repair activity [33, 36-39], namely, a systolic blood pressure ≥ 140mmHg and/or a diastolic blood pressure ≥ 90mmHg. To avoid the influence of undernutrition and paralysis caused by stroke, subjects with low body mass index (BMI; < 18.5kg/m^2^) (*n* = 45) and history of stroke (*n* = 42) were also excluded from the analysis, as were persons with missing data (*n* = 6 for handgrip data and *n* = 3 for CIMT data). The remaining participants, comprising 795 subjects (451 men and 344 women) with a mean age of 73.0 years (standard deviation (SD): 7.4; range: 60-89), were enrolled in the study.

This study was approved by the Ethics Committee for Human Use of Nagasaki University (project registration number 14051404). Written consent forms were available in Japanese to ensure comprehensive understanding of the study objectives, and informed consent was provided by the participants.

### Data collection and laboratory measurements

Body weight and height were measured with an automatic body composition analyzer (BF-220; Tanita, Tokyo, Japan) and BMI (kg/m^2^) was calculated. Systolic and diastolic blood pressure were recorded at rest.

Blood samples were collected in an EDTA-2K tube and a siliconized tube. Platelet levels in samples from the EDTA-2K tube were measured at SRL, Inc. (Tokyo, Japan) using an automated procedure.

Triglycerides (TG) and creatinine were each measured enzymatically. HDL-cholesterol (HDL) was measured using the direct method, while hemoglobin A1c (HbA1c) was measured using the latex coagulation method.

Handgrip strength was recorded as the grip strength from 2 measurements performed with each hand using a handgrip dynamometer (Smedley, Matsumiya Ika Seiki Seisakujo, Tokyo, Japan), with the maximum value used.

Measurement of carotid intima media thickness (CIMT) was determined by ultrasonography of the left and right common carotid arteries by an experienced vascular technician using a LOGIQ Book XP with a 10-MHz transducer (GE Healthcare, Milwaukee, WI, USA). Mean and maximum values for the left and right CIMT were calculated using automated digital edge-detection software (Intimascope; MediaCross, Tokyo, Japan) and a protocol that has been described in detail elsewhere [40]. The values of right and left CIMT without measurement of plaque were calculated, and the max CIMT value was used for analysis. Since a previous study reported normal CIMT value as < 1.1mm, we defined atherosclerosis as CIMT ≥ 1.1mm [41].

### Statistical analysis

Characteristics of the study population based on handgrip strength and stratified by platelet levels were expressed as mean ± standard deviation.

Odds ratios (OR) and 95% confidence intervals (CI) for carotid atherosclerosis (CIMT ≥ 1.1mm) associated with handgrip strength were calculated with the aid of logistic regression models. In addition, subjects were stratified by platelet levels, since they may act as an indicator of vascular repair activity [33, 36].

Two different approaches were used for making adjustments for confounding factors. In the first, we adjusted only for sex-and age. In the second, we included other potential confounding factors, namely, smoking status, alcohol consumption, systolic blood pressure (mmHg), BMI (kg/m^2^), HDL-cholesterol (mg/dL), TG (mg/dL) and HbA1c (%). All statistical analyses were performed with the SAS system for Windows (version 9.3; SAS Inc., Cary, NC), with values of *p* < 0.05 regarded as being statistically significant.
